# Body Fat and Visceral Fat Values in Spanish Healthcare Workers: Associated Variables

**DOI:** 10.3390/nu17040649

**Published:** 2025-02-11

**Authors:** Pedro Javier Tárraga Marcos, Ángel Arturo López-González, Emilio Martínez-Almoyna Rifá, Hernán Paublini Oliveira, Cristina Martorell Sánchez, Pedro Juan Tárraga López, José Ignacio Ramírez-Manent

**Affiliations:** 1 Sant Joan University Hospital, 03550 Alicante, Spain; pedrojav2003@gmail.com; 2 ADEMA-Health Group of the University Institute for Research into Health Sciences (IUNICS) of the Balearic Islands, 07120 Palma de Mallorca, Spain; emilio@udemax.com (E.M.-A.R.); h.paublini@eue.edu.es (H.P.O.); c.martorell@eua.edu.es (C.M.S.); jignacioramirez@telefonica.net (J.I.R.-M.); 3 Faculty of Odontology, University School ADEMA-UIB, 07009 Palma de Mallorca, Spain; 4 Health Service of the Balearic Islands, 07003 Palma de Mallorca, Spain; 5 Faculty of Medicine, Castilla la Mancha University, 02071 Albacete, Spain; pjtarraga@sescam.jccm.es; 6 Faculty of Medicine, Balearic Islands University, 07122 Palma de Mallorca, Spain

**Keywords:** obesity, body fat, visceral fat, Mediterranean diet, physical activity, healthcare workers

## Abstract

Background/Objectives: Excessive body adiposity is a significant public health challenge on a global scale. This study aimed to investigate the association between various sociodemographic factors and healthy lifestyle habits and the presence or absence of elevated body adiposity levels. Methodology: Two studies were conducted, a retrospective longitudinal study and a cross-sectional descriptive study. The analysis included 44,939 healthcare workers, categorised into four professional groups, to explore the relationship between age, sex, smoking, physical activity, and adherence to the Mediterranean diet and body adiposity, assessed as elevated body fat (BF) and visceral fat (VF) levels. Descriptive statistics encompassed categorical and quantitative variables, analysed using frequencies, Student’s *t*-tests, chi-square tests, and multinomial logistic regression models. Associations, concordances, and correlations were further examined using logistic regression and Cohen’s and Pearson’s kappa coefficients. Results: Age, sex, and physical activity were the factors most strongly associated with elevated BF and VF levels. Odds ratios (ORs) indicated the following significant associations: individuals aged 60 years and older exhibited ORs of 6.71 (95% CI: 5.68–7.74) for BF and 12.18 (95% CI: 10.01–14.26) for VF; male sex was associated with ORs of 2.21 (95% CI: 2.06–2.36) for BF and 12.51 (95% CI: 11.29–13.74) for VF. Sedentary behaviour was linked to ORs of 3.69 (95% CI: 3.41–3.97) for BF and 4.20 (95% CI: 3.78–4.63) for VF. Among healthcare professionals, nursing assistants and orderlies demonstrated the highest levels of adipose tissue accumulation. Conclusions: Elevated BF and VF levels among healthcare personnel are significantly associated by lifestyle factors, sex, and age, with the most pronounced risk observed in nursing assistants and orderlies. Further research focusing on the causal relationships between lifestyle behaviours and adiposity in this population will provide valuable insights and support the design of targeted preventive strategies to mitigate its prevalence.

## 1. Introduction

Excessive accumulation of adipose tissue in the body, commonly referred to as body adiposity, has become a major public health concern and is considered by many international health organisations as one of the leading pandemics of the 21st century [1]. This condition encompasses both subcutaneous fat [2], located beneath the skin, and visceral fat [3], which surrounds the internal organs within the abdominal cavity. Visceral fat is particularly significant due to its association with various chronic diseases [3].

From a physiological perspective, adipose tissue plays critical roles beyond serving as an energy reservoir [4], functioning as a key endocrine and metabolic regulator [5]. Visceral adipose tissue, situated in the abdominal cavity, is especially impactful due to its link to chronic low-grade inflammation [6] and the development of insulin resistance [7], making it a critical factor in metabolic and cardiovascular risk [8].

Over recent decades, the prevalence of body adiposity has risen at an alarming rate, reaching epidemic proportions globally [9]. According to data from the World Health Organisation (WHO), nearly 2 billion adults were classified as overweight, with more than one-third categorised as obese [10]. This phenomenon has also affected children and adolescents, with exponential increases in obesity and overweight rates in recent decades [11]. The main contributors to this trend include a combination of genetic, behavioural, and environmental factors, such as physical inactivity, excessive caloric intake, and sleep disturbances [12].

The clinical implications of body adiposity are diverse and complex. From a cardiovascular standpoint, visceral fat accumulation is associated with hypertension [13], dyslipidaemia [14], and an increased risk of coronary heart disease [15]. Additionally, this condition contributes to the development of metabolic syndrome [16], which encompasses central obesity, hyperglycaemia, hypertension, and dyslipidaemia, significantly increasing the risk of type 2 diabetes and cardiovascular diseases [17]. Regarding neoplastic diseases, evidence links obesity to an elevated risk of developing cancers such as colorectal cancer [18], breast cancer in postmenopausal women [19], and pancreatic cancer [20]. Further, a bidirectional association between obesity and mental health disorders, including depression [21] and anxiety [22], has also been observed.

Accurate assessment tools are essential for evaluating body adiposity and its distribution. While the body mass index (BMI) is widely used due to its simplicity, it has significant limitations, as it does not differentiate between lean and fat mass or reflect adipose tissue distribution [23]. In this context, alternative indicators such as waist circumference [24] and the Clínica Universitaria de Navarra Body Adiposity Estimator (CUN BAE) [25] have gained prominence. Advanced imaging techniques, including computed tomography (CT) [26] and magnetic resonance imaging (MRI) [27], allow for the precise quantification of visceral and subcutaneous fat, though their cost and availability may restrict routine use.

There are valid, cost-effective methods for assessing both total body fat and visceral fat, with bioelectrical impedance analysis (BIA) as one of the most prominent. This method measures the electrical resistance of different body tissues by applying a low-intensity alternating current, thereby estimating the body composition. BIA stands out as a simple, quick, and harmless technique, making it particularly useful in clinical settings. Its low cost and accessibility make it a practical tool for medical consultations and population studies. It is especially effective for evaluating the body composition in healthy individuals, those with normal weight, and also those with moderate overweight or obesity. Additionally, as a non-invasive method, it is ideal for repeated use in medical follow-ups, contributing to the monitoring and management of conditions related to excess body fat [28,29].

The management of body adiposity requires a comprehensive approach that integrates lifestyle modifications, pharmacological interventions, and, in selected cases, surgical options. The foundation of initial treatment lies in promoting a balanced diet [30] and incorporating regular physical activity [31]. Studies have demonstrated that even moderate weight loss of 5–10% can lead to significant improvements in metabolic and cardiovascular health [32]. When behavioural changes are insufficient, pharmacological agents, such as GLP-1 receptor agonists, have shown efficacy in weight reduction [33]. For severe obesity or cases with significant comorbidities, bariatric surgery offers an effective therapeutic option, providing sustained benefits in weight loss and the improvement of associated conditions [34].

In conclusion, body adiposity is a multifaceted condition that demands a holistic understanding and an interdisciplinary approach. Exploring its definition, epidemiological impact, clinical complications, diagnostic tools, and therapeutic options is essential to combat this global epidemic. Advances in scientific knowledge, coupled with the implementation of effective public health strategies, are critical to mitigating the adverse consequences of body adiposity on population health.

The aim of this study was to analyse how sociodemographic variables, such as age, sex, and socioeconomic status, as well as health habits, including smoking, adherence to the Mediterranean diet, and physical activity, are associated with levels of body fat and visceral fat as determined by bioelectrical impedance in a cohort of healthcare workers.

## 2. Materials and Methods

### 2.1. Participants

This research employed two studies, integrating a retrospective longitudinal analysis with a cross-sectional descriptive study. The study included a total of 44,939 healthcare professionals from various regions across Spain. Among the participants, 14,305 were men (31.8%) while 30,634 were women (68.2%). The sample was derived from individuals undergoing mandatory annual health evaluations required by their employers during the study period. The retrospective longitudinal study was conducted on a sample of workers for whom data on the studied variables (age, occupational category, smoking status, physical activity, and adherence to the Mediterranean diet) were available in 2010 and for whom the same variables were also available in 2019. The analysis aimed to assess how these variables influenced body fat and visceral fat levels under real-life conditions in this population. These are considered real-life conditions because no specific intervention was conducted by the researchers; instead, any observed changes were influenced by routine medical care from primary care physicians, social group interventions, personal decisions, and the influence of family and friends, among other factors. By comparing the same population over time, individuals who were under 30 years old in 2010 would be between 30 and 39 years old in 2019. However, for the purpose of comparison, they were still classified within the under-30 age group in 2019, as each age group was compared to itself a decade later. The same methodology was applied to the remaining variables.

For the cross-sectional study, the same sample of patients was used, but only data from 2019 were analysed.

Inclusion Criteria:

- Individuals between 18 and 69 years of age.
- Employment in one of the collaborating organisations.
- Provision of informed consent to participate in the study.
- Authorisation for the use of their data for epidemiological analysis.

Exclusion Criteria:

- Individuals younger than 18 or older than 69 years.
- Lack of an employment relationship with a participating organisation.
- Failure to provide informed consent for study participation.
- Refusal to authorise the use of personal data for epidemiological research.

**Figure 1:**
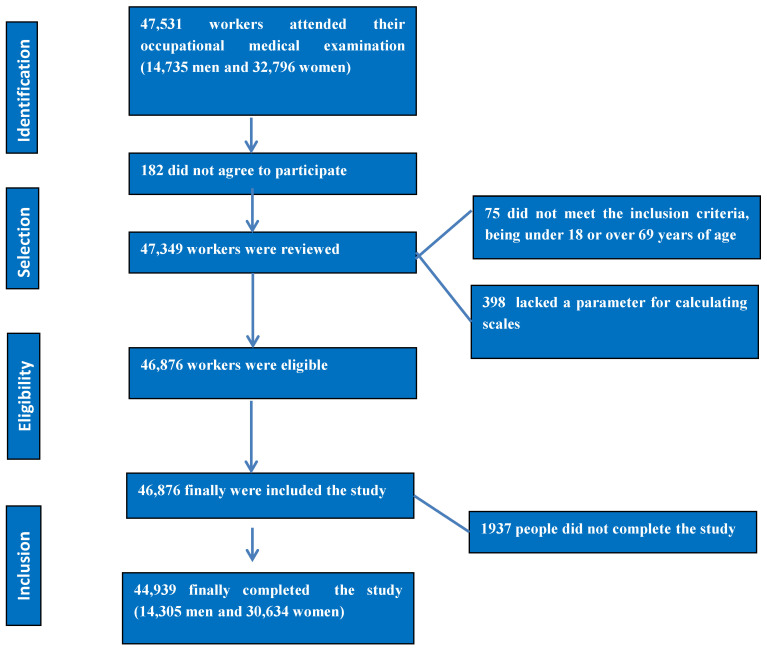
A flowchart detailing the selection and inclusion process for study participants.

The participant selection process is summarised in Figure 1.

### 2.2. Determination of Variables

Data collection was carried out by occupational health teams from participating organisations using the following structured methodology:

- Medical History: Information related to sociodemographic characteristics (e.g., age, sex, and professional role) and health behaviours—such as smoking status, physical activity patterns, and adherence to the Mediterranean diet—was obtained.
- Physical and Clinical Assessments: Anthropometric and physiological parameters (including height, weight, waist and hip circumferences, and systolic and diastolic blood pressure) were documented.
- Laboratory Testing: Biochemical analyses focused on lipid profiles, markers of liver function, and fasting glucose concentrations.

#### 2.2.1. Anthropometric Determinations

To mitigate potential biases, all of the measurements were performed with the following rigorously standardised protocols:

- Height and Weight: Participants’ height and weight were measured while standing, wearing only underwear, with their arms at their sides and their head and chest aligned. A SECA 700 scale and a SECA 220 stadiometer (SECA, Chino, CA, USA) were used for the measurements. The procedures followed the international standards for anthropometric assessment established by ISAK [35].
- Circumferences: Waist circumference was measured using a SECA tape (SECA, Chino, CA, USA), positioned midway between the iliac crest and the lowest rib, while hip circumference was recorded at the widest part of the buttocks. All of the measurements were taken with participants in an upright and relaxed posture.
- Body and Visceral Fat Assessment: Body fat and visceral fat levels were measured using bioelectrical impedance analysis with the Tanita DC 430MA device (TANITA Corporation, Tokyo, Japan). Elevated visceral fat was defined as values of 10 or higher based on the bioimpedance scale, while thresholds for high body fat varied according to the age-specific criteria provided by the device. The Gallagher classification was used to classify this percentage [36].

#### 2.2.2. Clinical Determinations

- Blood Pressure: Measurements were conducted using an OMRON-M3 sphygmomanometer (OMRON, Osaka, Japan) after participants had rested in a seated position for at least 10 min. Participants were instructed to abstain from consuming food, beverages, or tobacco for a minimum of one hour prior. Three readings were taken at one-minute intervals, and the average was calculated.

#### 2.2.3. Analytical Determinations

Venous blood samples were collected following a 12-h fasting period. Samples were refrigerated and transported to certified laboratories for analysis within 72 h. The following parameters were evaluated:

- Triglycerides, Total Cholesterol, and Glucose: Assessed using enzymatic methods.
- High-Density Lipoprotein (HDL) Cholesterol: Determined through a precipitation-based approach.
- Low-Density Lipoprotein (LDL) Cholesterol: Calculated using the Friedewald equation [37] for triglyceride levels below 400 mg/dL. For levels greater than 400 mg/dL, LDL was determined directly.

#### 2.2.4. Risk Scales

- Professional Categories: Participants were grouped into the following four professional roles: physicians, nurses, health technicians (e.g., laboratory, pathology, and radiology), and nursing assistants or orderlies.
- Smoking Definition: An individual was classified as a smoker if they had regularly consumed at least one cigarette per day (or its equivalent in other forms of tobacco use) within the prior month or had ceased smoking within the previous year.
- Dietary Assessment: Adherence to the Mediterranean diet (MD) was evaluated using the “Mediterranean Diet Adherence Questionnaire,” a tool derived from the PREDIMED test. The questionnaire comprises 14 items, each scored as either 0 or 1 point. A total score of less than 9 was categorised as low adherence, while scores of 9 or higher were indicative of good adherence [38].
- Physical Activity Assessment: Physical activity levels were measured using the International Physical Activity Questionnaire (IPAQ). This self-administered instrument consists of seven items designed to capture the type, duration, and frequency of physical activities undertaken in daily life over the preceding seven days [39,40]. The physical activity questionnaires (IPAQ) were completed during the clinical interview conducted on the day of the occupational medical examination. The health professionals supervised the proper completion of the questionnaire,

### 2.3. Statistical Analysis

Descriptive statistics for the categorical variables were summarised using frequencies and percentage distributions. The Kolmogorov–Smirnov test was conducted to evaluate the normality of continuous variables, followed by the computation of means and standard deviations. For the bivariate analysis, Student’s *t*-test was employed to compare group means, while the chi-square test was used to evaluate differences in proportions.

Factors associated with body fat and visceral fat were examined using multinomial logistic regression models. The goodness-of-fit of the logistic model was assessed via the Hosmer–Lemeshow test. Stratified analyses were conducted to identify possible confounding variables; however, no significant confounders were identified. Correlation and agreement between body fat and visceral fat were measured using Pearson’s correlation coefficient and Cohen’s kappa statistic. All of the statistical analyses were performed with SPSS software (version 29.0), adopting a significance level of 0.05.

## 3. Results

Table 1 provides an overview of the anthropometric, clinical, biochemical, sociodemographic, and lifestyle characteristics of the 44,939 healthcare professionals included in the study. The participants’ mean age was slightly above 41 years, with most individuals between the ages of 30 and 49. Across all of the variables, women demonstrated lower mean values compared to men.

Adherence to the MD was reported by 45.8% of male participants and 37.9% of female participants, whereas regular physical activity was carried out by 47.5% of men and 38.9% of women. The prevalence of smoking was marginally higher in men, with 16.1% of male participants and 15.0% of female participants reporting tobacco use.

Table 2 presents the mean values of body fat and visceral fat levels according to age, the occupational category among healthcare workers, smoking status, physical activity, and adherence to the MD in both sexes. Both the mean values and prevalence of elevated levels of these types of fat increased with age and decreased as the socioeconomic status improved. Higher values were also observed among smokers, sedentary individuals, and workers with a low adherence to the Mediterranean diet.

Mean body fat levels were higher in women, whereas the prevalence of elevated body fat levels was greater in men. Meanwhile, both the mean values and prevalence of elevated visceral fat levels were lower in women. In all cases, the differences observed were statistically significant (*p* < 0.001).

**Figure 2:**
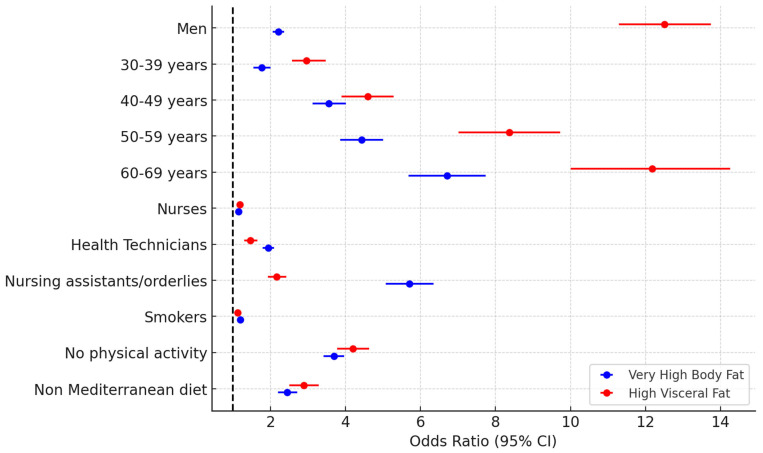
Forest Plot of the association between sociodemographic variables, healthy habits, and the professional category of healthcare workers and very high body fat and high visceral fat.

Figure 2 summarises the findings of the multinomial logistic regression analysis. All of the independent variables incorporated into the model, including age, sex, professional category, smoking status, physical activity, and adherence to the MD, were significantly associated with the occurrence of very high body fat levels and elevated visceral fat levels. Among these factors, age, sex, and physical activity demonstrated the strongest associations, as reflected by their respective odds ratios.

Pearson’s correlation coefficient between body fat and visceral fat was 0.512, while Cohen’s kappa statistic between very high body fat and high visceral fat was 0.529.

Table 3 summarises the findings of the retrospective longitudinal study conducted between 2010 and 2019. The results indicate that differences in the prevalence of very high body fat levels and elevated visceral fat values between the two time periods increased with age. A similar upward trend is observed with decreasing socioeconomic status. Both of the prevalences were higher among smokers, individuals with a sedentary lifestyle, and those who did not consistently adhere to a Mediterranean diet.

## 4. Discussion

Body adiposity, characterised by increased body fat and visceral fat percentages, is a complex, multifactorial condition associated in our research to variables such as age, sex, socioeconomic status (SES), smoking habits, dietary patterns, and physical activity levels. Healthcare workers represent a unique subset of the population due to the nature of their occupational environment, which includes a range of additional workplace stressors that can negatively impact lifestyle habits. These factors include the constant need to update knowledge; dealing with death, pain, and the suffering of others; public scrutiny of their work; the adoption of new technologies; and challenges within the public healthcare system, such as budget cuts, staff shortages, shift work, and excessive workloads. These stressors may contribute to the development of obesity. Understanding the interaction between these factors and body adiposity is essential for designing interventions to address obesity within this and other occupational groups [41].

Our findings indicate that age and sex are among the most strongly non-modifiable factors associated with body adiposity. Consistently, several studies have reported a positive relationship between aging and increased adiposity [42,43]. Older individuals tend to exhibit higher BMI and body fat percentages, driven by age-related physiological changes such as a reduced basal metabolic rate [44], increased fat accumulation, particularly visceral fat [45,46], and sarcopenia secondary to aging. This condition is further exacerbated by physical inactivity, endocrine alterations, chronic diseases, inflammation, insulin resistance, and inadequate nutrition [47].

Research within healthcare workers corroborates these trends. Studies, including those conducted by our team and by Vasquez-Purí et al., found that older Spanish healthcare professionals had a significantly greater prevalence of obesity compared to younger groups, with male workers more susceptible to overweight and obesity than females [48,49]. This sex difference may stem from hormonal factors [50] and distinct fat distribution patterns, as men tend to accumulate more visceral fat, whereas women primarily store peripheral fat [51]. Additionally, gender-specific cultural and occupational expectations may exacerbate disparities. Female healthcare workers, for instance, often juggle dual professional and domestic roles, potentially limiting their time and resources for maintaining healthy lifestyles and increasing their vulnerability to adiposity [52,53].

SES, represented in our study by the professional categories of healthcare workers, was strongly associated with body adiposity. Similar findings have been reported across various occupational groups, linking lower SES to limited access to nutritional foods, opportunities for physical activity, and healthcare resources. These disparities are particularly evident among healthcare workers in lower professional strata, such as nursing assistants and orderlies, compared to physicians or administrative staff [49,54,55].

The EpiDoC cohort study underscores the relationship between SES and obesity, highlighting the fact that individuals in lower socioeconomic strata had poorer adherence to the Mediterranean diet—a dietary pattern known for its protective effects against obesity [56]. Among healthcare workers, those with lower SES are more likely to adopt unhealthy eating habits due to financial constraints and irregular work schedules [57]. Additionally, lower SES has been associated with higher occupational stress levels, which may exacerbate weight gain through stress-induced eating behaviours [58].

The association between smoking and body adiposity, clearly observed in our study, is a topic of complexity in the literature. Smoking has traditionally been linked to lower body weight due to appetite suppression [59,60], and smoking cessation often results in significant weight gain [61]. This is particularly relevant in populations with a high prevalence of smoking, such as healthcare workers. A systematic review and meta-analysis examining smoking habits among healthcare professionals reported a slightly higher smoking prevalence among males and those in lower professional categories [62]. Stress and shift work, common in healthcare settings, may increase tobacco use as a coping mechanism. Meanwhile, other studies have linked smoking to increased visceral fat accumulation [63], an effect that is particularly pronounced during menopause [64]. Our results revealed an association between smoking and increased levels of both body and visceral fat, with high statistical significance (*p* < 0.001). This underscores the role of smoking as a modifiable risk factor for body adiposity.

Our findings highlight the protective association of adherence to the MD against body adiposity. The MD emphasises the consumption of fruits, vegetables, whole grains, olive oil, and moderate amounts of fish and poultry, while limiting processed foods and red meat. Studies have shown an inverse relationship between adherence to the MD and BMI or body fat percentage. For example, research on dietary patterns in a Spanish working population revealed significantly lower rates of obesity and central adiposity among individuals with higher adherence to the MD [65]. However, among healthcare workers, adherence to this dietary pattern is often hindered by irregular work schedules and limited access to healthy food options during shifts [66]. Workplace initiatives, such as providing Mediterranean-style meals supervised by a nutritionist in hospital cafeterias, could effectively promote healthier dietary habits within this group. The PREDIMED study, a landmark trial on the MD, demonstrated its efficacy in reducing cardiovascular risk factors, including abdominal obesity [67].

Our study confirms the strong association between physical activity and body adiposity. These results are consistent with previous research, showing that regular exercise improves body composition by reducing fat mass and preserving lean muscle mass [68,69,70]. The IPAQ (International Physical Activity Questionnaire) has been widely used to assess physical activity levels in working populations. Studies using the IPAQ found that healthcare workers engaging in regular physical activity exhibited significantly lower BMI and body fat percentages compared to their sedentary counterparts. Conversely, sedentary behaviour, often exacerbated by prolonged hours in clinical or administrative roles, contributes to increased adiposity [71]. In our study, we found a strong association between physical inactivity and increased total body fat and visceral fat, with odds ratios of 3.69 and 4.20, respectively. We believe this is a significant contribution, as it provides a more accurate assessment of body fat quantity and distribution compared to BMI.

Workplace wellness programs designed to promote physical activity—such as onsite fitness facilities, group exercise classes, or incentivised participation in physical activity challenges—have shown promise in addressing these issues. Randomised controlled trials and retrospective cohort studies conducted in hospital settings reported significant reductions in BMI and waist circumference among staff participating in workplace fitness programs [72,73]. Encouraging healthcare workers to incorporate physical activity into their daily routines, even through simple measures like walking or stretching during breaks, could significantly impact reducing adiposity.

Healthcare workers are particularly vulnerable to occupational stress and irregular work schedules, both of which are independently associated with increased body adiposity. This may be linked to the activation of the hypothalamic–pituitary–adrenal (HPA) axis by stress, leading to elevated cortisol levels that promote fat accumulation, particularly in the visceral region [74,75,76,77]. This phenomenon has been observed among medical students in Panama, as well as in other populations [78].

Shift work disrupts circadian rhythms, negatively affecting metabolic processes and contributing to weight gain [79]. A systematic review of shift work and obesity highlighted the increased risk of central obesity among shift workers compared to those with regular work schedules [80]. Healthcare professionals, particularly nurses and emergency staff [81], often experience disrupted sleep patterns and irregular meal schedules, further exacerbating the risk of adiposity [82].

Addressing these challenges requires systemic changes, such as implementing evidence-based shift scheduling practices and providing resources for stress management, including counselling programs and mindfulness training [83,84].

Body image plays a fundamental role in the lives of individuals with obesity, influencing their emotional and social well-being as well as their ability to adopt healthy lifestyle habits. A negative perception of one’s own body can lead to feelings of dissatisfaction and rejection, directly affecting self-esteem and psychological well-being. Many individuals with obesity experience a deteriorated self-concept, which translates into a higher prevalence of anxiety, depression, and eating disorders. This dissatisfaction with body image can create a vicious cycle in which frustration and stress promote unhealthy behaviours, such as physical inactivity or the consumption of ultra-processed foods, as an emotional coping mechanism, thereby perpetuating obesity [85].

Socialisation is also profoundly affected by body image in individuals with obesity. Weight-related discrimination and stigma create barriers to social interaction, potentially leading to social isolation and difficulties in establishing interpersonal relationships. Weight stigma is prevalent in multiple settings, including educational, professional, and healthcare environments, impacting self-confidence and personal development. In many cases, fear of rejection or feelings of shame discourage participation in group activities, such as sports or social events, thereby limiting opportunities to adopt healthier behaviours and reinforcing physical inactivity.

In the workplace, obesity can affect employment opportunities and professional development. Various studies have shown that individuals with obesity are less likely to access well-paid jobs with favourable working conditions. Additionally, they often face biases in hiring and promotion processes, as obesity is erroneously associated with a lack of discipline or lower productivity. These economic disadvantages may further limit access to healthy food choices and resources that promote well-being, such as gym memberships or specialised medical treatments, perpetuating obesity and its negative consequences.

Ultimately, the quality of life of individuals with obesity is compromised by these interconnected factors. Body dissatisfaction, social isolation, occupational barriers, and emotional distress create an environment that hinders adherence to healthy lifestyle habits. To break this cycle, it is essential to implement multidisciplinary intervention strategies that address obesity from a biopsychosocial perspective, promoting body acceptance, nutritional education, and equitable access to health and wellness opportunities [86]. In our study, we were unable to assess this concept, since the participants in it were people who attend the mandatory annual health check-up of different companies. This check-up does not include the evaluation of self-perception of body image.

This multidimensional approach underscores the importance of addressing the various factors influencing body adiposity in healthcare workers. Tailored interventions targeting these factors could lead to significant improvements in their overall health and well-being.

The importance of this study lies in raising awareness and understanding the impact of non-communicable diseases on healthcare personnel, a key group whose health directly affects the well-being of the entire population. The stressors inherent in the healthcare professionals’ work environment create a unique combination of risk factors that significantly contribute to the physical and emotional burnout of these workers. In many cases, this exhaustion leads them to adopt unhealthy lifestyle habits as a coping mechanism.

It is crucial to recognise that protecting and improving the health of this group is not only a matter of labour justice but also a strategic necessity for the healthcare system. Public health policies and healthcare service managers must prioritise the development of effective measures to prevent the adoption of harmful habits and promote healthy lifestyles among healthcare professionals. Promoting health within this group directly benefits the workers, increases their well-being and productivity, and improves the quality of care provided to patients. Further, a healthy workforce strengthens the efficiency of the healthcare system and ultimately contributes to the overall well-being of the population.

Among the strategies considered necessary to improve healthcare personnel’s health, several concrete actions are highlighted. First, proper health education training for all personnel working in healthcare settings to foster the adoption of positive habits. Second, the implementation of nutritional policies in the workplace, such as offering healthy meals in hospital cafeterias supervised by nutritionists, and eliminating vending machines, which promote the consumption of ultra-processed foods. Third, ensuring work schedules that allow for proper meal times, along with the installation of physical fitness areas within the workplace. Finally, the development of stress management programs, such as mindfulness, yoga, and relaxation techniques, is essential to reduce the psychological impact of the work environment.

These actions would not only improve the health of healthcare personnel but also ensure the long-term sustainability and quality of healthcare systems.

## 5. Strengths and Limitations

This study boasts several strengths, including its extensive sample size of nearly 45,000 healthcare workers, positioning it as one of the largest investigations conducted globally in this population. Additionally, it is among the pioneering studies to evaluate body adiposity, specifically body fat and visceral fat measured through bioimpedance, across diverse roles within the healthcare sector. The comprehensive analysis of variables—spanning sociodemographic factors and lifestyle characteristics—paired with a longitudinal design facilitates the identification of causal relationships.

Another notable advantage is the implementation of validated questionnaires for assessing physical activity levels and adherence to the Mediterranean diet. These tools offer a practical, cost-efficient, and reliable approach for evaluation and follow-up purposes.

Nevertheless, this study is not without limitations.

The main limitation of the study is the non-inclusion as a variable of body image, which some authors have considered as a possible mediator in the relationship between excessive adiposity and physical inactivity and unhealthy eating habits.

Another limitation of the study is that it excludes certain groups, such as unemployed individuals, retirees, and those younger than 18 or older than 69 years of age, which may restrict the generalisability of the findings to the wider population. However, the large sample size likely offsets this limitation to some extent.

Moreover, as the participants were exclusively from Spain, the findings may not be directly applicable to other populations. As a result, these outcomes should be interpreted with caution when considering their applicability to different demographic or geographic groups.

An additional limitation stems from the reliance on self-administered questionnaires. This method is inherently subject to certain biases, including recall bias and the influence of social desirability. Future studies could enhance the reliability of findings by integrating objective validation methods.

Lastly, the study did not account for certain potential confounders, such as the presence of comorbidities or the use of pharmacological treatments, as this information was unavailable for analysis.

## 6. Conclusions

Body adiposity in healthcare workers is associated with a complex interaction of factors, such as age, sex, socioeconomic status, smoking, dietary patterns, and physical activity. These determinants could be aggravated by the specific challenges faced by healthcare professionals, such as work stress and irregular working hours, which have not been assessed in this study. Therefore, in order to address this problem, a multifaceted approach incorporating individual, organisational, and societal interventions may be necessary. Strategies such as promoting adherence to the Mediterranean diet, encouraging regular physical activity, and addressing stress through workplace wellness programs could be instrumental in mitigating adiposity in this population. Future research should focus on longitudinal studies to better understand the causal pathways and efficacy of interventions aimed at reducing obesity among healthcare workers.

## Figures and Tables

**Table 1:** Characteristics of the population.

	Men n = 14,305	Women n = 30,634	
	Mean (SD)	Mean (SD)	*p*-Value
Age (years)	41.1 (10.6)	40.4 (10.5)	<0.001
Height (cm)	176.0 (7.5)	162.6 (6.0)	<0.001
Weight (kg)	81.2 (14.5)	63.7 (13.3)	<0.001
Waist circumference (cm)	89.7 (12.6)	76.7 (11.8)	<0.001
Hip circumference (cm)	101.7 (8.8)	99.3 (10.7)	<0.001
Systolic blood pressure (mmHg)	128.2 (13.1)	116.1 (13.8)	<0.001
Diastolic blood pressure (mmHg)	79.9 (10.6)	74.8 (10.1)	<0.001
Total cholesterol (mg/dL)	191.8 (37.2)	187.8 (34.6)	<0.001
HDL-c (mg/dL)	48.9 (11.2)	59.3 (12.8)	<0.001
LDL-c (mg/dL)	165.2 (46.2)	144.8 (38.9)	<0.001
Triglycerides (mg/dL)	111.0 (73.2)	81.7 (47.0)	<0.001
Glucose (mg/dL)	93.6 (18.2)	88.9 (12.4)	<0.001
AST (U/L)	24.1 (17.2)	18.2 (8.0)	<0.001
ALT (U/L)	29.0 (36.7)	17.3 (13.7)	<0.001
**GGT (U/L)**	30.2 (28.8)	18.1 (18.1)	<0.001
	**n (%)**	**n (%)**	***p*** **-Value**
<30 years	2400 (16.8)	5984 (19.5)	<0.001
30–39 years	4200 (29.4)	8304 (27.1)	
40–49 years	4512 (31.5)	10,128 (33.0)	
50–59 years	2449 (17.1)	5150 (16.8)	
60–69 years	744 (5.2)	1120 (3.6)	
Physicians	5064 (35.4)	5024 (16.4)	<0.001
Nurses	4008 (28.0)	12,752 (41.6)	
Health technicians	1728 (12.1)	4128 (13.5)	
Nursing assistants or orderlies	3505 (24.5)	8782 (28.5)	
Non-smokers	12,001 (83.9)	26,094 (85.0)	<0.001
Smokers	2304 (16.1)	4592 (15.0)	
No physical activity	7512 (52.5)	18,744 (61.1)	<0.001
Yes physical activity	6793 (47.5)	11,942 (38.9)	
No Mediterranean diet	7771 (54.2)	19,213 (62.1)	<0.001
Yes Mediterranean diet	6534 (45.8)	11,413 (37.9)	

HDL: high-density lipoprotein. LDL: low-density lipoprotein. AST: aspartate aminotransferase. ALT: alanine aminotransferase. GGT: gamma-glutamyl transpeptidase. SD: standard deviation.

**Table 2:** Mean values of percentage of body and visceral fat according to sociodemographic variables and health habits.

		Body Fat		Visceral Fat	
Men	n	Mean (SD)	*p*-Value	Mean (SD)	*p*-Value
<30 years	2400	15.1 (7.1)	<0.001	3.4 (3.0)	<0.001
30–39 years	4200	18.0 (6.3)		5.9 (3.6)	
40–49 years	4512	20.2 (8.3)		8.4 (3.7)	
50–59 years	2449	24.1 (6.6)		12.0 (4.0)	
60–69 years	744	27.5 (6.4)		13.1 (3.8)	
Physicians	5064	19.0 (7.0)	<0.001	7.5 (4.6)	<0.001
Nurses	4008	18.0 (7.5)		6.2 (4.4)	
Health technicians	1728	21.7 (10.2)		8.5 (4.6)	
Nursing assistants or orderlies	3505	21.8 (7.7)		9.2 (4.7)	
Non-smokers	12,001	19.5 (8.0)	<0.001	7.5 (4.8)	<0.001
Smokers	2304	21.1 (7.3)		7.7 (4.4)	
No physical activity	7512	22.5 (7.9)	<0.001	9.2 (4.8)	<0.001
Yes physical activity	6793	16.7 (6.7)		6.0 (3.9)	
No Mediterranean diet	7771	21.7 (7.7)	<0.001	8.9 (4.5)	<0.001
Yes Mediterranean diet	6534	17.5 (6.8)		6.4 (3.5)	
**Women**	**n**	**Mean (SD)**	***p*** **-Value**	**Mean (SD)**	***p*** **-Value**
<30 years	5984	24.9 (6.6)	<0.001	2.1 (2.2)	<0.001
30–39 years	8304	26.9 (7.4)		3.3 (2.5)	
40–49 years	10,128	30.0 (7.6)		5.1 (2.7)	
50–59 years	5150	32.8 (7.6)		7.1 (2.8)	
60–69 years	1120	33.5 (7.1)		8.2 (3.4)	
Physicians	5024	25.0 (6.7)	<0.001	3.1 (2.6)	<0.001
Nurses	12,752	27.2 (7.1)		3.7 (2.6)	
Health technicians	4128	31.3 (7.8)		5.2 (3.1)	
Nursing assistants or orderlies	8782	31.9 (7.9)		6.1 (3.5)	
Non-smokers	26,094	28.6 (7.7)	<0.001	4.4 (3.1)	<0.001
Smokers	4592	29.6 (8.4)		5.0 (3.4)	
No physical activity	18,744	30.2 (7.9)	<0.001	4.9 (3.5)	<0.001
Yes physical activity	11,942	26.4 (7.1)		3.7 (2.5)	
No Mediterranean diet	19,213	28.8 (7.4)	<0.001	4.7 (3.4)	<0.001
Yes Mediterranean diet	11,413	27.0 (7.5)		3.9 (2.6)	

SD: standard deviation.

**Table 3:** Differences in the prevalences of very high values of body fat and high values of visceral fat between the pre- and post-periods by sex.

		Very High BF			High VF		
Men	n	%Pre–Post	% Difference	*p*-Value	%Pre–Post	% Difference	*p*-Value
<30 years	2400	5.7–6.0	5.3	<0.001	2.8–3.0	7.1	<0.001
30–39 years	4200	9.8–10.6	8.2		5.2–5.7	9.6	
40–49 years	4512	10.8–12.1	12.0		10.0–11.7	17.1	
50–59 years	2449	20.8–24.5	17.8		32.3–38.2	18.3	
60–69 years	744	20.4–25.8	26.5		45.3–58.1	28.3	
Physicians	5064	7.0–7.1	1.2	<0.001	11.9–12.6	5.9	<0.001
Nurses	4008	11.1–12.0	8.0		8.3–9.0	8.4	
Health technicians	1728	12.1–13.9	14.9		14.4–16.7	16.0	
Nursing assistants or orderlies	3505	19.2–24.0	25.1		17.7–21.9	23.7	
Non-smokers	12,001	11.4–12.4	8.8	<0.001	13.8–15.2	10.1	<0.001
Smokers	2304	16.9–18.8	11.2		14.2–16.7	17.6	
No physical activity	7512	16.0–20.1	25.6	<0.001	17.4–22.4	28.7	<0.001
Yes physical activity	6793	5.6–6.0	7.1		7.3–7.8	6.8	
No Mediterranean diet	7771	15.2–18.5	21.7	<0.001	16.0–20.1	25.6	<0.001
Yes Mediterranean diet	6534	7.3–7.9	8.2		8.5–9.3	9.4	
**Women**	**n**	**%**		***p*** **-Value**	**%**		***p*** **-Value**
<30 years	5984	2.8–2.9	3.6	<0.001	0.3–0.3	3.7	<0.001
30–39 years	8304	5.4–5.8	7.4		1.0–1.2	11.5	
40–49 years	10,128	9.1–10.1	11.0		1.6–1.9	12.8	
50–59 years	5150	14.4–16.4	11.6		3.7–4.3	16.2	
60–69 years	1120	17.8–21.4	20.2		9.4–11.4	21.3	
Physicians	5024	1.5–1.6	6.7	<0.001	0.5–0.6	5.6	<0.001
Nurses	12,752	5.0–5.4	8.0		0.8–1.0	14.3	
Health technicians	4128	13.7–15.3	11.7		3.4–3.9	17.3	
Nursing assistants or orderlies	8782	13.7–16.3	19.0		3.4–4.0	20.2	
Non-smokers	26,094	8.2–8.9	8.5	<0.001	1.8–2.0	11.1	<0.001
Smokers	4592	8.7–9.8	12.6		3.0–3.5	15.8	
No physical activity	18,744	10.3–12.3	19.4	<0.001	2.9–3.5	20.7	<0.001
Yes physical activity	11,942	3.6–3.9	8.3		0.3–0.4	11.9	
No Mediterranean diet	19,213	9.8–11.5	17.4	<0.001	2.6–3.1	19.2	<0.001
Yes Mediterranean diet	11,413	4.6–4.9	6.5		0.5–0.6	10.5	

BF: body fat. VF: visceral fat. Pre-year 2010. Post-year 2019.

## Data Availability

This study’s data are stored in a database that complies with all security measures at the ADEMA-Escuela Universitaria. The Data Protection Delegate is Ángel Arturo López González.
